# Rückgang der Anwendung hormoneller Kontrazeptiva bei Frauen: Genese kritischer Einstellungen in reproduktiven Biografien: Ergebnisse der Studie „frauen leben 4 – Familienplanung im Lebenslauf“

**DOI:** 10.1007/s00103-026-04211-z

**Published:** 2026-03-06

**Authors:** Sabrina Mannebach, Laura Olejniczak, Sonja Glaser, Tilmann Knittel

**Affiliations:** 1 Sozialwissenschaftliches Forschungsinstitut zu Geschlechterfragen – SoFFI F., Forschungs- und Innovationsverbund an der Evangelischen Hochschule Freiburg (FIVE e. V.), Bugginger Straße 38, 79114 Freiburg, Deutschland; 2 Cornelia Helfferich Institut für Geschlechter- und Familienforschung, Freiburg, Deutschland

**Keywords:** Hormonelle Verhütung, Pille, Agency, Kontrazeption, Beratung, Hormonal contraception, Pill, Agency, Contraception, Counselling

## Abstract

**Hintergrund:**

Die Nutzung der Pille, des meistgenutzten hormonellen Kontrazeptivums, ist in Deutschland seit mehreren Jahren rückläufig. Ziel der Untersuchung ist es, die aktuelle Entwicklung der Anwendung der Pille nach Alter differenziert darzustellen und aus biografischer Perspektive ein vertieftes Verständnis der Entstehung kritischer Einstellungen zur Pille und zu hormonellen Kontrazeptiva insgesamt zu gewinnen.

**Methoden:**

Analysiert wurden Daten zur Anwendung und Bewertung der Pille aus der Mixed-Methods-Studie „frauen leben 4“ mit 5579 Frauen mit Verhütungsbedarf. Die Ergebnisse wurden mit Daten der Vorgängerstudie von 2012 verglichen. Zudem wurden 20 qualitative Interviews zu kritischen Einstellungen gegenüber hormonellen Kontrazeptiva inhaltsanalytisch und hermeneutisch-rekonstruktiv ausgewertet.

**Ergebnisse:**

Die Pillenanwendung ging von 45,7 % im Jahr 2012 auf 25,3 % im Jahr 2024 zurück. Kritische Einstellungen sind bei jüngeren Befragten stärker verbreitet. Ihre Entwicklung wird begünstigt durch unzureichende informationsbasierte Entscheidungsmöglichkeiten, insbesondere zu Beginn der Verhütungsbiografie, was Agency und Selbstbestimmung einschränkt. Die Distanzierung von hormoneller Verhütung erfolgt als reflexiver Prozess. Zwischen kritischer Einstellung gegenüber hormonellen Kontrazeptiva und ihrer Nichtnutzung besteht ein enger Zusammenhang.

**Diskussion:**

Der Rückgang der Pillenanwendung steht im Einklang mit den Befunden anderer Studien. Die Interviews zeigen unzureichend gedeckte Informationsbedarfe zur hormonellen Kontrazeption sowie daraus resultierende Einschränkungen der Selbstbestimmung bei Verhütungsentscheidungen. Dies unterstreicht die Bedeutung bedarfsorientierter Aufklärung und Beratung für selbstbestimmtes reproduktives Handeln.

**Zusatzmaterial online:**

Zusätzliche Informationen sind in der Online-Version dieses Artikels (10.1007/s00103-026-04211-z) enthalten.

## Abstract (en)

**Background:**

The use of the pill, the most widely used hormonal contraceptive, has been declining in Germany for several years. The aim of the study is to present current trends in pill use differentiated by age and to gain a more detailed understanding of the development of critical attitudes towards the pill and hormonal contraceptives in general from a biographical perspective.

**Methods:**

Data were analysed on the use and evaluation of the pill from the mixed-methods study “women’s lives 4” involving 5579 women with contraceptive needs. The results were compared with data from the previous 2012 study. In addition, 20 qualitative interviews on critical attitudes towards hormonal contraceptives were evaluated using content analysis and hermeneutic reconstruction.

**Results:**

Pill use declined from 45.7% in 2012 to 25.3% in 2024. Critical attitudes are more prevalent among younger respondents. Their development is facilitated by insufficient information-based decision-making options, especially at the beginning of the contraceptive biography, which limits agency and self-determination. The distancing from hormonal contraception takes place as a reflexive process. There is a close connection between critical attitudes towards hormonal contraceptives and their non-use.

**Discussion:**

The decline in pill use is consistent with the findings of other studies. The interviews reveal insufficiently met information needs regarding hormonal contraception and the resulting limitations on self-determination in contraceptive decisions. This underscores the importance of needs-based education and counselling for self-determined reproductive behaviour.

## Einleitung

Die Nutzung der „Pille“^1^ ist in Deutschland seit Jahren rückläufig [1–3]. Parallel dazu ist eine zunehmende Verbreitung kritischer Einstellungen gegenüber hormonellen Kontrazeptiva beobachtbar [1, 4–6]. Aufschlüsse zum Rückgang der Pillennutzung sind relevant, da diese bis 2018 die am häufigsten angewendete Kontrazeptionsmethode in Deutschland war [1] und ihre Methodensicherheit hoch ist [7]. Bei bisherigen Forschungen stehen die Bewertung der Verträglichkeit und Sorge vor Nebenwirkungen zugeführter Hormone auf Psyche und Sexualität [1, 4–6, 8, 9] im Vordergrund. Als Einflüsse für die zunehmende Verbreitung kritischer Einstellungen werden die lange Debatte um potenzielle Nebenwirkungen besonders der Pille [9–11], ein gestiegenes Gesundheitsbewusstsein gerade bei jungen Menschen [2] sowie mediale Einflüsse diskutiert [12].

Ziel dieser explorativ angelegten Untersuchung ist es, ein genaueres Verständnis der Entwicklung kritischer Einstellungen zur Pille und zu hormonellen Kontrazeptiva allgemein im Verlauf der Verhütungsbiografie zu gewinnen – und damit auch zur rückläufigen Nutzung der Pille sowie deren Folgen.

Die vorliegende Arbeit ergänzt bisherige Forschungen um eine biografische Perspektive [13]. In Anlehnung an Helfferich wird *Verhütung als biografisches, reproduktives Handeln* verstanden, das auf biografischem Wissen basiert, gesellschaftlich gerahmt ist und sich prozesshaft über Entscheidungen, Anwendung und mögliche Methodenwechsel in den Biografien sequenziell entfaltet [14]. Verhütungsentscheidungen entstehen nicht als rein individuelle Präferenzen, sondern in biografisch, historisch und sozial strukturierten Möglichkeitsräumen, die von Geschlechterverhältnissen, Normen und institutionellen Praktiken – etwa der Verschreibungspraxis – geprägt sind. Gesellschaftliche Alters- und Sequenzregeln normieren reproduktives Handeln und bestimmen das richtige (Mindest‑)Alter und weitere biografische Voraussetzungen (z. B. „erst Ausbildung, dann Kind“) für eine Familiengründung. Dies führt bei Frauen in jüngerem Alter zu hohen Anforderungen an die Sicherheit der Verhütungsmethoden [13]. Vor diesem Hintergrund ist es zielführend, die *Agency*^2^ der Frauen einzubeziehen.

Der Untersuchung liegt ein Mixed-Methods-Design zugrunde. Mit der Frage, wie sich kritische Einstellungen zu hormonellen Kontrazeptiva im biografischen Verlauf entwickeln, liegt der Schwerpunkt der vorgestellten Ergebnisse auf den explorativen *qualitativen Analysen*. Die biografische Perspektive ermöglicht Aufschlüsse darüber, in welchen biografischen Phasen kritische Einstellungen gegenüber hormoneller Kontrazeption ausgebildet werden und wie sich dieser Prozess im Kontext der Erfahrungsaufschichtung [15] und des normativen Rahmens reproduktiver Biografien gestaltet. Die statistischen Datenanalysen dienen zur Einordnung der Befunde der qualitativen Analysen und hierbei insbesondere zum Zusammenhang von kritischen Einstellungen, der tatsächlichen Nutzung der Pille und der Verhütungspraxis insgesamt.

## Methoden

Die Auswertungen basieren auf Daten der vom Bundesinstitut für Öffentliche Gesundheit^3^ finanziell geförderten Studie „frauen leben 4 – Familienplanung im Lebenslauf“, die vom Sozialwissenschaftlichen Forschungsinstitut zu Geschlechterfragen (SoFFI F.) an der Evangelischen Hochschule Freiburg von 2023 bis 2025 durchgeführt wurde.

### Repräsentative Befragung und statistische Auswertungen

Die quantitative Datenbasis von „frauen leben 4“ bildet eine repräsentative Befragung von *N* = 7111 im Juni und Juli 2024 befragten Frauen im Alter von 20 bis 44 Jahren aus den Bundesländern Baden-Württemberg, Berlin, Niedersachsen und Sachsen. Als Stichprobe wurde in einem 2‑stufigen Verfahren eine Zufallsauswahl von Einwohnermeldeamtsadressen gezogen. Insgesamt wurden 24.750 Frauen der Altersgruppe postalisch zur Teilnahme eingeladen. Die Befragung, die ein breites Themenspektrum zu Familienplanung, sozialer Situation, Geburten, Schwangerschaftsabbrüchen und Verhütung umfasste, erfolgte mittels Online-Selbstausfüllerbogen. Die technische und administrative Umsetzung der Befragung erfolgte durch das Institut infas GmbH in Bonn. Die inhaltliche Entwicklung des Fragebogens sowie die statistischen Analysen wurden vom SoFFI F. durchgeführt. Die teilnehmenden Frauen erhielten im Nachgang ein Incentive. Die Rücklaufquote der Befragung lag bei 28,7 %.

Die Datensätze wurden nach den Kriterien bundeslandspezifische Bevölkerungszahl, Altersgruppe, Haushaltsgröße und höchster allgemeinbildender Schulabschluss auf Grundlage des Mikrozensus 2023 gewichtet. Sämtliche Auswertungen in diesem Artikel beziehen sich auf die Teilgruppe der heterosexuell aktiven Frauen ohne aktuellen Kinderwunsch, die sich selbst und ihre Sexualpartner als fertil einschätzen (*n* = 5579). Bei diesen Frauen kann ein Verhütungsbedarf angenommen werden und im Falle der Nichtanwendung von Verhütungsmethoden ein ungedeckter Verhütungsbedarf, hinter dem verschiedene Motivlagen stehen können [19]; Tab. 1 zeigt die Stichprobenbeschreibung.

**Tab. 1:** Soziodemografische Merkmale der Stichprobe. Die Prozentangaben beziehen sich auf die gewichteten Daten. Ausgewiesen sind Frauen mit Verhütungsbedarf. (Datenquelle: Studie „frauen leben 4“)

	Anteil in % (ungewichtetes *n*, gewichtetes *n*)
*Gesamt*	100 (5579, 5504)
*Alter*	
20 bis 24 Jahre	17,9 (934, 983)
25 bis 29 Jahre	19,2 (1128, 1058)
30 bis 34 Jahre	20,9 (1102, 1152)
35 bis 39 Jahre	21,1 (1195, 1159)
40 bis 45 Jahre	21,0 (1220, 1153)
*Bildung*	
Niedrig	12,9 (195, 713)
Mittel	28,8 (1311, 1585)
Hoch	58,3 (4073, 3206)
*In fester Beziehung/verheiratet*	
Ja	80,5 (4546, 4432)
Nein	19,5 (1033, 1072)
*Kinder*	
Keine Kinder	52,3 (3037, 2876)
1 Kind	15,7 (885, 865)
2 oder mehr Kinder	32,0 (1657, 1763)
*Bundesland*	
Baden-Württemberg	41,7 (1550, 2298)
Berlin	16,4 (1135, 902)
Niedersachsen	28,8 (1488, 1583)
Sachsen	13,1 (1406, 721)

Für einen Zeitvergleich zur Anwendung der Pille werden zusätzlich Daten der Studie „frauen leben 3“ herangezogen, bei der im Jahr 2012 insgesamt *n* = 4002 Frauen (darunter *n* = 2927 mit Verhütungsbedarf) im Alter zwischen 20 und 44 Jahren in den gleichen 4 Bundesländern telefonisch befragt worden sind [14].

Die statistischen Auswertungen wurden mit SPSS 29 (IBM, Armonk, NY, USA) durchgeführt. Sie umfassen deskriptive bivariate Auswertungen und Zusammenhangsanalysen. Zusammenhänge wurden mittels Chi^2^-Tests geprüft und deren Stärke mit Cramer V ausgewiesen. Das Signifikanzniveau wurde auf α = 0,05 festgelegt. Prozentangaben in Tabellen werden mit 95 %-Konfidenzintervallen berichtet.

### Qualitative Interviews und Analyse

Die 20 Teilnehmerinnen der qualitativen Teilstudie wurden kriteriengeleitet nach Alter und mittels folgender 3, an Befragungen des BIÖG zum Verhütungsverhalten Erwachsener [1] angelehnten Screeningitems zur kritischen Einstellung gegenüber der Anwendung der Pille in der Repräsentativbefragung ausgewählt:

- „Verhütung mit der Pille hat negative Auswirkungen auf Körper und Seele“,
- „Verhütung mit der Pille kann man unbedenklich über Jahre hinweg anwenden“,
- „Für mich persönlich kommt es wegen der Nebenwirkungen nicht (mehr) in Frage, mit der Pille zu verhüten“.

Die Antwortkategorien waren hierbei jeweils: 1 „stimme vollkommen zu“/2 „stimme eher zu“/3 „teils-teils“/4 „stimme eher nicht zu“/5 „stimme überhaupt nicht zu“. Das genaue Vorgehen bei der Auswahl der Interviewpartnerinnen ist in der Beschreibung des qualitativen Samples in Tab. 2 dargelegt. Bei der Auswahl der Teilnehmerinnen wurde systematisch auf die Variierung nach dem Bundesland sowie weiteren, in der Samplebeschreibung ausgewiesenen soziodemografischen Merkmalen geachtet. Eingeschlossen wurden ausschließlich Frauen mit zum Befragungszeitpunkt gedecktem oder ungedecktem Verhütungsbedarf.

**Tab. 2:** Sample der qualitativen Teilstudie (*n* = 20; aus der Studie „frauen leben 4“), Personen mit kritischer Einstellung gegenüber der Anwendung der Pille

Alter	Anzahl	Einstellung zur Anwendung der Pille^a^	Anzahl	Anwendung der Pille	Anzahl
20 bis unter 25	6	Starke Ablehnung	4	Nicht mehr, aber früher	2
				Noch nie	2
		Gemäßigte Ablehnung/Bedenken	2	Aktuelle Anwendung	2
25 bis unter 35	7	Starke Ablehnung	2	Nicht mehr, aber früher	2
		Gemäßigte Ablehnung/Bedenken	5	Nicht mehr, aber früher	3
				Noch nie	1
				Aktuelle Anwendung	1
35 bis unter 45	7	Starke Ablehnung	5	Nicht mehr, aber früher	5
		Gemäßigte Ablehnung/Bedenken	2	Nicht mehr, aber früher	2

Im Sample eingeschlossen waren dabei: *nach Bundesland*: *n* = 5 Befragte aus Baden-Württemberg, *n* = 4 aus Berlin, *n* = 6 aus Niedersachsen, *n* = 5 aus Sachsen;

*nach Bildungsstand*: *n* = 8 hoch, *n* = 5 höher, *n* = 6 mittel, *n* = 1 niedrig;

*nach Parität*: *n* = 11 kinderlose Frauen, *n* = 9 Frauen mit leiblichen Kindern;

*nach Migrationshintergrund*: *n* = 16 ohne Migrationshintergrund, *n* = 4 mit Migrationshintergrund

^a^ Als starke Ablehnung wurde gewertet, wenn im standardisierten Fragebogen die Fragen: „Für mich persönlich kommt es wegen der Nebenwirkungen nicht (mehr) in Frage, mit der Pille zu verhüten“ und „Verhütung mit der Pille hat negative Auswirkungen auf Körper und Seele“, mit „stimme vollkommen zu“ und gleichzeitig die Frage: „Verhütung mit der Pille kann man unbedenklich über Jahre hinweg anwenden“, mit „stimme überhaupt nicht zu“ beantwortet wurden. Als gemäßigte Ablehnung wurden alle weiteren Fälle gewertet, bei denen den ersten beiden Fragen teilweise, eher oder vollkommen zugestimmt und die dritte Frage teilweise, eher oder vollkommen abgelehnt wurde

Für die als narrative Interviews angelegte Erhebung wurde ein Leitfaden mit Erzählimpulsen [18, 20] entwickelt und im Rahmen von 4 Probeinterviews getestet und überarbeitet. Eine wesentliche Weiterentwicklung war die thematische Ausweitung von einer kritischen Einstellung gegenüber der Pille auf hormonelle Kontrazeptiva allgemein. Diese breitere Perspektive liegt auch anderen Studien zugrunde [1, 4–6]. Insgesamt wurden 20, durchschnittlich 45-minütige Interviews in Form von Online-Videotelefonaten geführt. Sämtliche Befragten wurden vor dem Interview datenschutzrechtlich aufgeklärt. Von allen befragten Frauen liegt eine Einwilligung gemäß Art. 13 EU-DSGVO vor. Die aufgezeichneten Interviews wurden transkribiert und anonymisiert.

#### Inhaltsanalyse.

In einem ersten Auswertungsschritt wurde eine inhaltlich strukturierende, kategorienbildende Inhaltsanalyse nach Kuckartz und Rädiker [21–23] mit dem Ziel durchgeführt, relevante Themenbereiche in diesem Komplex herauszuarbeiten. So konnte ein systematischer Überblick über thematische Schwerpunkte innerhalb der Interviews im biografischen Verlauf gewonnen werden. Die Kategorienbildung erfolgte deduktiv anhand der forschungsleitenden Fragen und wurde durch das Datenmaterial induktiv erweitert. Themenbezogene Fallzusammenfassungen und Kernaussagen der Analysen wurden zu Themenclustern gebündelt. Für das Forschungsinteresse zentrale Passagen oder Passagen mit erkennbarer Manifestation von Irritationen wurden für die sequenzielle Feinanalyse ausgewählt.

#### Sequenzielle Feinanalyse.

Um latente Sinnstrukturen und subjektive Sinngebungen in reproduktiven Biografien zu verstehen sowie zentrale Motive und Thematisierungsregeln der Befragten zu rekonstruieren, wurden an ausgewählten Textstellen sequenzielle Feinanalysen durchgeführt [24, 25, 31]. Grundlage ist das von Helfferich und Kruse [26] konzeptionierte und von Kruse [16] weiterentwickelte integrative Basisverfahren (IB), das ein rekonstruktiv-hermeneutisches Programm darstellt, welches sich diverser Ansätze wie der Wissenssoziologie Karl Mannheims und methodisch-methodologisch der Grounded Theory bedient [26]. Eine intersubjektive, sequenzielle Auswertung erfolgte in Interpretationsgruppen mit Fokus auf Analyseheuristiken wie der Agency‑, Metaphern- und Positioning-Analyse [16, 17]. Ziel war, das subjektive Erleben und Deutungsmuster zu hormonellen Kontrazeptiva sowie die Agency der interviewten Frauen in ihren Möglichkeitsräumen offen zu rekonstruieren. Mit der Agency-Analyse wurde die subjektive Wahrnehmung der „eigenen (Nicht‑)Beteiligung am Zustandekommen von Ereignissen“ herausgearbeitet [16]. Mithilfe der biografischen Perspektive kann Agency als veränderliche, sequenzielle Leistung in narrativen Kontexten reproduktiven Handelns innerhalb der Verhütungsbiografie rekonstruiert werden [13]. Das von Kruse beschriebene Vollprogramm wurde abgekürzt und für ausgewählte Textpassagen mit inhaltlichen Relevanzen oder sprachlichen Auffälligkeiten angewendet.

## Ergebnisse

### Quantitative Ergebnisse zur Anwendung und Beurteilung der Pille

**Abb. 1:**
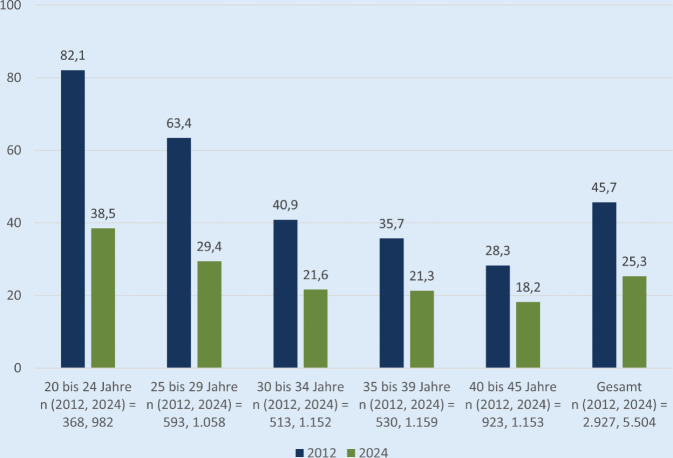
Anwendung der Pille nach Altersgruppen in den Erhebungsjahren 2012 und 2024 (Angaben in % bezogen auf Frauen mit Verhütungsbedarf). Datenquellen: Studien „frauen leben 3“ und „frauen leben 4“

Die Daten der „frauen-leben“-Studienreihe zeigen im Vergleich zwischen 2012 und 2024 einen deutlichen Rückgang der Pillenanwendung. Gaben in der Befragung 2012 altersübergreifend noch 45,7 % der Frauen mit Verhütungsbedarf an, die Pille zu nehmen, beträgt dieser Anteil bei der Befragung 2024 nur noch 25,3 % (Abb. 1). Der Rückgang der Pillenanwendung zeigt sich in sämtlichen Altersgruppen, ist bei jüngeren Frauen aber besonders stark. Wie aus Abb. 1 ebenfalls deutlich wird, ist der Anteil der Frauen, die mit der Pille verhüten, umso geringer, je älter die Frauen sind.

Ungeachtet der gesunkenen Anwendungshäufigkeit hat auch laut den Ergebnissen der aktuellen „frauen-leben-4“-Befragung eine Mehrheit der jüngeren Befragten die Pille bereits selbst angewendet (Tab. 3). Von den Geburtsjahrgängen 2000 bis 2004 wendeten 38,5 % der Frauen mit Verhütungsbedarf die Pille zum Befragungszeitpunkt an. Weitere 32,9 % hatten zu einem früheren Zeitpunkt mit der Pille verhütet und 28,4 % hatten keine eigene Erfahrung mit der Pille. Bei den älteren Geburtskohorten liegt der Anteil der Frauen ohne eigene Erfahrung mit der Pillenanwendung niedriger.

**Tab. 3:** Erfahrung mit und Einstellungen zu der Anwendung der Pille nach Geburtskohorten. Die Angaben beziehen sich auf Frauen mit Verhütungsbedarf. (Datenquelle: Studie „frauen leben 4“)

	Geburtskohorten						
	(Alter zum Befragungszeitpunkt)						
	*2000 bis 2004*	*1995 bis 1999*	*1990 bis 1994*	*1985 bis 1989*	*1979 bis 1984*	*V; p*	*Gesamt*
	(20 bis 24 Jahre)	(25 bis 29 Jahre)	(30 bis 34 Jahre)	(35 bis 39 Jahre)	(40 bis 44 Jahre)		*n* (ungewichtet, gewichtet) = 5579, 5504
	*n* (ungewichtet, gewichtet) = 934, 983	*n* (ungewichtet, gewichtet) = 1128, 1058	*n* (ungewichtet, gewichtet) = 1102, 1152	*n* (ungewichtet, gewichtet) = 1195, 1159	*n* (ungewichtet, gewichtet) = 1220, 1153		
	% [95 % KI]	% [95 % KI]	% [95 % KI]	% [95 % KI]	% [95 % KI]		% [95 % KI]
*Eigene Erfahrung mit der Anwendung der Pille*	–	–	–	–	–	0,188	–
						0,000	
Aktuelle Anwendung	38,5 [35,5; 41,6]	29,4 [26,7; 32,2]	21,6 [19,3; 24,1]	21,3 [19,0; 23,7]	18,2 [16,1; 20,5]	–	25,3 [24,2; 26,5]
Nicht mehr, aber früher	32,9 [30,0; 35,8]	53,9 [50,9; 56,9]	59,7 [56,9; 62,5]	60,1 [57,3; 62,9]	59,1 [56,3; 61,9]	–	53,8 [52,5; 55,1]
Noch nie	28,4 [25,6; 31,3]	15,7 [13,6; 18,0]	15,6 [13,6; 17,8]	9,6 [8,0; 11,4]	7,9 [6,4; 9,6]	–	15,0 [14,1; 16,0]
Keine Angabe	0,3 [0,1; 0,8]	0,9 [0,5; 1,7]	3,0 [2,2; 4,2]	9,0 [7,4; 10,7]	14,8 [12,8; 16,9]	–	5,9 [5,3; 6,5]
*Verhütung mit der Pille hat negative Auswirkungen auf Körper und Seele*	–	–	–	–	–	0,120	–
						0,000	
1–2 (Zustimmung)	73,8 [71,0; 76,5]	72,8 [70,1; 75,4]	71,7 [69,0; 74,2]	69,8 [67,1; 72,4]	54,8 [51,9; 57,7]	–	68,4 [67,1; 69,6]
3 (teils, teils)	19,6 [17,2; 22,1]	18,8 [16,6; 21,3]	19,6 [17,4; 21,9]	18,4 [16,3; 20,7]	26,2 [23,7; 28,8]	–	20,6 [19,5; 21,7]
4–5 (Ablehnung)	6,6 [5,2; 8,3]	8,3 [6,8; 10,1]	8,8 [7,3; 10,5]	11,8 [10,0; 13,7]	19,0 [16,8; 21,3]	–	11,1 [10,3; 11,9]
*Verhütung mit der Pille kann man unbedenklich über Jahre hinweg anwenden*	–	–	–	–	–	0,067	–
						0,000	
1–2 (Zustimmung)	12,2 [10,2; 14,3]	14,7 [12,7; 17,0]	18,0 [15,9; 20,3]	17,0 [14,9; 19,3]	21,1 [18,8; 23,5]	–	16,8 [15,8; 17,8]
3 (teils, teils)	20,6 [18,1; 23,2]	22,0 [19,6; 24,6]	20,9 [18,6; 23,3]	22,8 [20,5; 25,3]	24,5 [22,1; 27,0]	–	22,2 [21,1; 23,3]
4–5 (Ablehnung)	67,2 [64,3; 70,1]	63,2 [60,3; 66,1]	61,1 [58,3; 63,9]	60,2 [57,3; 63,0]	54,4 [51,5; 57,3]	–	61,0 [59,7; 62,3]
*Für mich persönlich kommt es wegen der Nebenwirkungen nicht (mehr) in Frage, mit der Pille zu verhüten*	–	–	–	–	–	0,071	–
						0,000	
1–2 (Zustimmung)	54,2 [51,1; 57,3]	59,2 [56,2; 62,1]	64,2 [61,4; 66,9]	67,9 [65,2; 70,6]	60,6 [57,7; 63,4]	–	61,5 [60,2; 62,8]
3 (teils, teils)	13,8 [11,8; 16,1]	11,4 [9,6; 13,4]	8,6 [7,1; 10,3]	7,9 [6,4; 9,5]	11,7 [10,0; 13,7]	–	10,6 [9,8; 11,4]
4–5 (Ablehnung)	31,9 [29,1; 34,9]	29,5 [26,8; 32,3]	27,2 [24,7; 29,8]	24,2 [21,8; 26,7]	27,7 [25,2; 30,3]	–	28,0 [26,8; 29,2]

Tab. 3 zeigt, dass kritische Einstellungen gegenüber der Pille bei jüngeren Befragten stärker verbreitet sind als bei älteren Jahrgängen. Knapp 3 Viertel der zwischen 1995 und 2004 geborenen Frauen teilen die Meinung, dass Verhütung mit der Pille negative Auswirkungen auf Körper und Seele hat. Insbesondere bei vor 1985 geborenen Frauen sind diese Vorbehalte mit einem Anteil von 54,8 % weniger verbreitet. Je älter die Befragten sind, desto häufiger stimmen sie auch der Aussage zu, dass die Pille über Jahre hinweg unbedenklich angewendet werden kann. Bei der Konsequenz für die Verhütungspraxis zeigt sich dagegen ein abweichendes Bild: Der Anteil der Frauen, die die Nutzung der Pille für sich aufgrund von Nebenwirkungen ausschließen, ist bei den jüngeren Frauen unter 25 Jahre am geringsten (54,2 %) und nimmt mit steigendem Alter bis zur Gruppe der 35- bis 39-jährigen Frauen (67,2 %) zu.

Je kritischer die Einstellungen zur Pille sind, desto seltener wird sie angewendet – dieser Zusammenhang zeigt sich bei allen 3 erhobenen Items (Tab. 4). Frauen mit ausgeprägt kritischer Einstellung zur Pille verhüten überdurchschnittlich häufig mit Kondom, zyklusbasierten Verhütungsmethoden sowie der Kupferspirale. Der Anteil der Frauen, die trotz Bedarf keine Verhütungsmethode anwenden, ist bei kritischer Einstellung zur Pille und Ablehnung ihrer Anwendung erhöht: Mit einem Anteil von 9,1 % ist der ungedeckte Verhütungsbedarf bei Frauen, die eine Anwendung der Pille wegen der Nebenwirkungen für sich entschieden ablehnen, signifikant höher als bei Frauen mit weniger starker Ablehnung (Cramer V = 0,124, *p* = 0,000). Eine kritische Einstellung zur Pille führt nicht durchgängig zum Verzicht auf ihre Anwendung: Frauen, die von negativen Auswirkungen der Pille auf Körper und Seele ausgehen, nutzen sie zu 16,5 %.

**Tab. 4:** Aktuelle Verhütungsmethoden nach Alter und nach Einstellungen zur Anwendung der Pille. Die Angaben beziehen sich auf Frauen mit Verhütungsbedarf. (Datenquelle: Studie „frauen leben 4“)

	*n* (ungewichtet, gewichtet)	Pille	V; *p*	Kondom	V; *p*	Kupferspirale	V; *p*	Zyklusbasierte Methoden	V; *p*	Keine Verhütung	V; *p*
		% [95 % KI]		% [95 % KI]		% [95 % KI]		% [95 % KI]		% [95 % KI]	
*Gesamt*	5579, 5504	25,4 [24,2; 26,5]		45,8 [44,5; 47,1]		5,3 [4,7; 5,9]		9,7 [8,9; 10,5]		6,7 [6,1; 7,4]	
*Altersgruppe*	–	–	0,164	–	0,151	–	0,048	–	0,046	–	0,073
			0,000		0,000		0,012		0,020		0,000
20 bis 24 Jahre	934, 983	38,5 [35,5; 41,6]	–	53,2 [50,1; 56,3]	–	4,8 [3,6; 6,3]	–	8,9 [7,2; 10,8]	–	3,4 [2,4; 4,6]	–
25 bis 29 Jahre	1128, 1058	29,4 [26,7; 32,2]	–	51,4 [48,4; 54,4]	–	6,2 [4,9; 7,8]	–	10,6 [8,8; 12,6]	–	6,9 [5,5; 8,6]	–
30 bis 34 Jahre	1102, 1152	21,6 [19,3; 24,1]	–	50,0 [47,1; 52,9]	–	6,7 [5,3; 8,2]	–	11,2 [9,5; 13,1]	–	6,1 [4,8; 7,6]	–
35 bis 39 Jahre	1195, 1159	21,3 [19,0; 23,7]	–	43,1 [40,3; 46,0]	–	3,7 [2,7; 4,9]	–	10,1 [8,5; 11,9]	–	8,4 [6,9; 10,1]	–
40 bis 44 Jahre	1220, 1153	18,2 [16,1; 20,5]	–	32,8 [30,1; 35,5]	–	5,0 [3,9; 6,4]	–	7,5 [6,0; 9,1]	–	8,5 [7,0; 10,2]	–
*Verhütung mit der Pille hat negative Auswirkungen auf Körper und Seele*	–	–	0,320	–	0,165	–	0,063	–	0,141	–	0,065
			0,000		0,000		0,000		0,000		0,000
1–2 (Zustimmung)	3803, 3754	16,5 [15,3; 17,7]	–	51,0 [49,4; 52,6]	–	6,2 [5,5; 7,0]	–	12,4 [11,4; 13,5]	–	7,8 [7,0; 8,7]	–
3 (teils, teils)	1111, 1131	38,5 [35,7; 41,3]	–	38,6 [35,8; 41,5]	–	3,7 [2,7; 4,9]	–	4,2 [3,2; 5,5]	–	5,4 [4,2; 6,8]	–
4–5 (Ablehnung)	653, 608	56,1 [52,1; 60,0]	–	27,1 [23,7; 30,8]	–	2,5 [1,4; 3,9]	–	2,3 [1,3; 3,7]	–	3,0 [1,8; 4,5]	–
*Verhütung mit der Pille kann man unbedenklich über Jahre hinweg anwenden*	–	–	0,344	–	0,130	–	0,073	–	0,135	–	0,045
			0,000		0,000		0,000		0,000		0,003
1–2 (Zustimmung)	859, 920	49,0 [45,8; 52,3]	–	38,2 [35,1; 41,3]	–	2,8 [1,9; 4,0]	–	4,8 [3,5; 6,3]	–	4,2 [3,1; 5,7]	–
3 (teils, teils)	1195, 1219	39,9 [37,1; 42,6]	–	37,4 [34,7; 40,2]	–	3,6 [2,7; 4,8]	–	4,6 [3,5; 5,9]	–	6,9 [5,6; 8,4]	–
4–5 (Ablehnung)	3511, 3348	13,6 [12,5; 14,8]	–	51,0 [49,3; 52,7]	–	6,6 [5,8; 7,5]	–	12,8 [11,7; 14,0]	–	7,4 [6,5; 8,3]	–
*Für mich persönlich kommt es wegen der Nebenwirkungen nicht (mehr) in Frage, mit der Pille zu verhüten*	–	–	0,698	–	0,200	–	0,148	–	0,173	–	0,124
			0,000		0,000		0,000		0,000		0,000
1–2 (Zustimmung)	3536, 3380	2,5 [2,1; 3,1]	–	53,6 [51,9; 55,3]	–	7,9 [7,0; 8,8]	–	13,6 [12,5; 14,8]	–	9,1 [8,1; 10,1]	–
3 (teils, teils)	481, 581	36,7 [32,8; 40,6]	–	38,7 [34,8; 42,7]	–	1,9 [1,0; 3,3]	–	6,4 [4,6; 8,6]	–	5,9 [4,2; 8,0]	–
4–5 (Ablehnung)	1550, 1538	71,2 [68,9; 73,5]	–	31,5 [29,2; 33,8]	–	0,8 [0,5; 1,4]	–	2,2 [1,6; 3,0]	–	2,0 [1,4; 2,8]	–

Für eine vollständige Darstellung aller erhobenen Verhütungsmethoden siehe Tabelle Z1 im Onlinematerial

### Ergebnisse der qualitativen Teilstudie zur Genese von kritischen Einstellungen zu hormonellen Kontrazeptiva

Im Zentrum der qualitativen Analysen stand der Prozess der Entwicklung kritischer Einstellungen zu hormoneller Verhütung. Ziel war es, ein detailliertes Verständnis der subjektiven Motive und thematisch relevanten Felder bei dieser Entwicklung in den unterschiedlichen Phasen der reproduktiven Biografien zu gewinnen. Deutlich wurde eine ausgeprägte *Heterogenität kritischer Einstellungen*, wobei das Spektrum von strikter Ablehnung von Hormonen wie „Paranoia … gegenüber Hormonen“ (I 8) über ambivalente Haltungen bis hin zur (eingeschränkten) Akzeptanz reicht. Kritische Einstellungen zeigen sich bei Frauen mit, aber auch ohne eigene negative Erfahrungen mit hormoneller Verhütung. Auch die handlungspraktischen Konsequenzen sind vielfältig.

Als zentral bei der Genese kritischer Einstellungen erwies sich der *Zusammenhang von Information und Agency*. Inhaltsanalytisch konnten relevante Themenfelder in den verschiedenen Phasen der Verhütungsbiografie herausgearbeitet werden wie „Informationen“^4^, „Pille als Standardprogramm“, „Nebenwirkungen“, „Methodenwechsel“ oder „Hormone trotz Unerwünschtheit“. Feinanalytisch wurden subjektive Motive, Agency und Positionierungen wie Schuld und Scham, Vertrauensbruch, Unsicherheit, Distanzierung zum früheren Selbst und Alternativlosigkeit in den verschiedenen biografischen Sequenzen rekonstruiert.

**Abb. 2:**
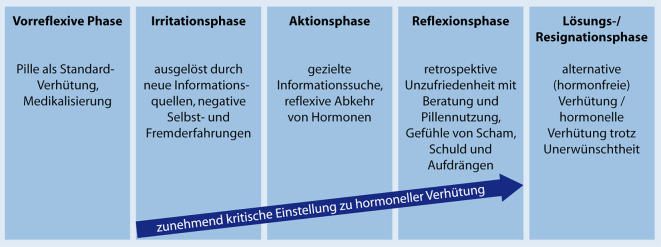
Modell der Genese kritischer Einstellung zu hormonellen Kontrazeptiva in der Verhütungsbiografie

Die Ergebnisse der qualitativen Analysen wurden in dem „Modell der Genese kritischer Einstellung zu hormonellen Kontrazeptiva in der Verhütungsbiografie“ (Abb. 2) zusammengeführt und verdichtet. Es besteht aus 5 Phasen:

#### Vorreflexive Phase

Die vorreflexive Phase markiert den Beginn der Verhütungsbiografie. Während der Adoleszenz und häufig vor dem ersten Geschlechtsverkehr findet ein konkreter Kontakt mit dem Thema Verhütungsmethoden meist zuerst in der gynäkologischen Praxis statt. Vorerst steht die Mehrheit der Befragten hormoneller Verhütung zu diesem Zeitpunkt nicht kritisch gegenüber. Die Frauen berichten, dass sie sich stark an ärztlichen Empfehlungen orientieren, ohne eigenes kritisches Hinterfragen und Reflektieren. „Hatte ich mich tatsächlich nie mit befasst“ (I 7).

Der erste Besuch bei der/dem Gynäkolog*in wird häufig als *kurz und uninformativ* beschrieben: „Also das war jetzt nicht so ’n sehr ausführliches Aufklärungsgespräch“ (I 10). Rückblickend wird der Entscheidungsprozess zur ersten Verhütung von den Frauen mit einer *fehlenden Agency *verbunden. Aspekte, die in diesen Beratungskontexten besonders zum Tragen kommen, sind kollektiv verankerte Deutungsmuster reproduktiven Handelns:

*Pille als Standardprogramm: *Die Pille wird laut den Befragten häufig routinemäßig ohne Abwägung von Alternativen verordnet und zugleich von den Frauen unhinterfragt genutzt: „Das war einfach so ein Standardprogramm. Weil irgendwie alle sich die Pille haben verschreiben lassen“ (I 10). Die Befragten berichten von einer unzureichenden Aufklärung in der gynäkologischen Beratungssituation: „Untersuchung und Rezept und tschüss“ (I 15). Rückblickend wird die Wahl der Verhütungsmethode vielfach als wenig selbstbestimmt und die Nutzung hormoneller Kontrazeptiva als unüberlegt beschrieben.

*Medikalisierung und die Pille: *Die Pille wird nicht nur zur Empfängnisverhütung verordnet und genutzt, sondern auch zur Zyklusregulation, zum Schmerzmanagement oder aus Schönheitsaspekten. Teils wird die Pille bereits Jahre vor dem ersten Geschlechtsverkehr jenseits der Verhütung eingesetzt. Die Ausweitung medizinischer Deutungs- und Behandlungslogiken der Pille auf körperliche und soziale Prozesse als Teil einer Medikalisierungsdynamik [10] führt zu einer frühen und langen Einnahmezeit, was im Verlauf der Verhütungsgeschichte wiederholt als Argument für eine kritische Haltung gegenüber hormoneller Verhütung angeführt wird.

#### Irritationsphase

In der Irritationsphase beginnt sich die kritische Einstellung zu entwickeln. Auslöser dafür sind vor allem der *Zugang zu zuvor unbekannten Informationen* zu negativen Nebenwirkungen. Hier ist zum einen die Selbsterfahrung mit negativen Nebenwirkungen zu nennen. Die Frauen erzählen wiederholt, dass diese Körpererfahrungen wie Stimmungsschwankungen, Depressionen, Libidoverlust, vaginale Atrophie etc. abweichend zu den Informationen aus den ersten Beratungsgesprächen in den gynäkologischen Praxen stünden. Zum anderen sind auch Berichte über negative Nebenwirkungen von Peers bzw. medial vermittelte Informationen über TV oder Social Media relevant in dieser Phase.

Der Zugang zu Informationen spielt eine wichtige Rolle bei der Veränderung der Einstellung zu hormonellen Kontrazeptiva. Dies führt zu einer *kognitiven Neubewertung* der Einnahme von Hormonen. Im Zuge der bewussten Wahrnehmung von Risiken und Nebenwirkungen fühlen sich die Frauen von ärztlicher Seite nicht ausreichend beraten und sehen ihre Handlungsmacht eingeschränkt: „Wieso hat denn das VORHER niemand gesagt?“ (I 15).

#### Aktionsphase

In der Aktionsphase steigt die Kritik weiter an und die Pille wird häufig abgesetzt. Die erlebte Dissonanz zwischen gynäkologischer (Erst‑)Beratung und negativen Erfahrungen führt zu einer *bewussten Entscheidung gegen Hormone* und einer aktiven Suche nach detaillierten, auch wissenschaftlich fundierten Fachinformationen zu Risiken hormoneller Verhütung über „seriöse Quellen“ (I 3) wie Fachinformationsseiten. Dies erhöht die Agency: „Also habe ich mich im Endeffekt selbst informiert und selbst beraten“ (I 13). Die komplexen Wirkungsweisen von Hormonen bleiben zugleich häufig intransparent. Überforderung und Unsicherheit entstehen, was auf ein verbleibendes Wissensdefizit hinweist. Auf der Suche nach alternativen Verhütungsoptionen suchen viele Frauen zudem wiederholt Rat in ihrer gynäkologischen Praxis. Vielfach wird berichtet, dass Sorgen dort keine Resonanz gefunden haben: „Und da bin ich echt bei Frauenärzten ÜBERHAUPT nicht ernstgenommen worden“ (I 13). Dies kann als Delegitimierung ihrer Empfindungen gelesen werden, was Unsicherheiten bezüglich der Einnahme von Hormonen weiter begünstigt und den Vertrauensverlust gegenüber der medizinischen Beratung manifestiert.

#### Reflexionsphase

In der Reflexionsphase findet ein bewusstes Nachdenken über die Hormoneinnahme und die Situation des ersten Beratungsgesprächs statt. Viele Befragte äußern retrospektiv den Eindruck, ihnen sei zu Beginn der Verhütungsbiografie hormonelle Verhütung *aufgedrängt* worden: „Ansonsten wurde man gefühlt eher in eine Entscheidung hineingedrängt“ (I 12). Die rückblickend als unzureichend empfundene Beratung wird mit einer passiven Agency verbunden: „Also ich weiß noch, dass … da mehr oder weniger über mich entschieden wurde“ (I 9). Das *Informationsdefizit,* aufgrund dessen Verhütungsentscheidungen getroffen wurden, kann zu einem Bedauern der Nutzung hormoneller Verhütung führen. Die Verantwortung, uninformiert in die Erstberatung gegangen zu sein und dem eigenen Körper lange Hormone zugeführt zu haben, wird häufig internalisiert. Schuld und Scham sowie eine Distanzierung vom uninformierten früheren Selbst sind die Folge.

#### Lösungs‑/Resignationsphase

Im Verlauf der Verhütungsbiografie kann es zu einer Stagnation der kritischen Einstellung kommen. In der finalen Phase des Modells treffen Frauen eine abwägende, *reflexive Entscheidung* für eine Verhütungsmethode. Diese kann in der Wahl hormonfreier Verhütungsmethoden oder in der Akzeptanz hormoneller Kontrazeptiva bestehen: Teils werden *Hormone trotz Unerwünschtheit* genutzt. Genannt werden Aspekte wie der Mangel an sicheren oder zur Familienplanung passenden Alternativen: „Risiko oder Hormone“ (I 1), die Anwendungsfreundlichkeit sowie häufig auch das Schmerzmanagement: „Pest oder Cholera, Schmerz oder Depression“ (I 18). In dieser letzten Phase wird ein Kontrast zwischen der ersten Verhütungserfahrung als bevormundet, unreflektiert, uninformiert und der späteren Verhütungsentscheidung als informationsbasiert, reflektiert und abwägend ersichtlich. Die Handlungsspielräume der als aktiv rekonstruierten Agency sind zugleich aufgrund individueller und gesellschaftlicher Rahmenbedingungen eingeschränkt. „Da kennt man sich dann schon mit sehr vielen Wirkstoffen aus und ja des war dann für uns beide schon sehr kritisch, aber es ist die einfachste und in DEM Falle auch MIT sicherste Variante“ (I 4).

## Diskussion

Die ausgeführte statistische Analyse zeigt übereinstimmend mit anderen Erhebungen [1, 4, 6] einen starken Rückgang der Nutzung der Pille und einen engen Zusammenhang zwischen einer kritischen Einstellung zur Pille und ihrer Nichtnutzung. In der Lebenslaufperspektive nimmt Verhütung mit der Pille mit fortschreitendem Alter ab, wobei dies nicht ausschließlich auf kritische Einstellungen zurückführen ist. Verhütung ist als Prozess biografischer Entscheidungen in unterschiedlichen Abschnitten des Lebenslaufs im Zusammenhang mit Aspekten wie der Lebensphase, Partnerschaft, Alter oder Kinderwunsch zu betrachten [13, 14].

Hervorzuheben ist, dass der Wechsel von der Pille zu anderen Verhütungsmethoden anders als noch 12 Jahre zuvor eine jüngere Alterskohorte betrifft. Die „frauen-leben-4“-Daten zeigen, dass 71 % der 20- bis 24-jährigen Frauen mit aktuellem Verhütungsbedarf in ihrem Leben bereits die Pille genutzt haben – allerdings nutzen sie aktuell lediglich noch 38 %. Es ist anzunehmen, dass die Phasen des qualitativ entwickelten Modells immer früher und schneller durchlaufen werden. Auf die Verbreitung kritischer Einstellungen bereits bei Jugendlichen verweist die Jugendsexualitätsstudie [4]. Ein denkbarer Einflussfaktor ist die Zunahme von digitalen Informationsmöglichkeiten [12].

Mit der biografischen Perspektive konnte in der qualitativen Analyse im „Modell der Genese kritischer Einstellung zu hormonellen Kontrazeptiva in der Verhütungsbiografie“ das reproduktive Handeln in seinem Bezug zu den Erfahrungen und Möglichkeitsräumen rekonstruiert und relevante Themenfelder im biografischen Verlauf expliziert werden. Als zentrales Ergebnis ist die *Bedeutung von Information* im biografischen Verlauf festzuhalten. Informationen werden sowohl in Form von negativen Selbsterfahrungen sowie in Form von (medialen) Berichten über negative Fremderfahrungen mit Nebenwirkungen relevant gemacht.

Es besteht eine starke *Interdependenz zwischen Information, Agency und der Bewertung hormoneller Kontrazeptiva*: Fehlt zu Beginn der Verhütungsbiografie eine informationsbasierte Entscheidungsmöglichkeit, sind die Agency und infolge die Selbstbestimmung^5^ eingeschränkt, was die Ausbildung einer kritischen Haltung gegenüber hormonellen Kontrazeptiva begünstigt. Die Befragten bewerten die gynäkologische Beratung retrospektiv mehrheitlich als unzureichend und stark auf die Pille ausgerichtet. Entsprechend eingeschränkt handlungsmächtig rekonstruieren die Frauen ihre reproduktiven Entscheidungen für die Pille zu Beginn der Verhütungsbiografie. Dies kann zu Verunsicherung und zum Verlust des Vertrauens in medizinische Beratung führen. Nebenwirkungen werden von den Frauen vor allem unter den Aspekten fehlender Informationen thematisiert. Das heißt, dass Gesundheit als Argument für eine kritische Einstellung [1, 4–6, 8, 9] eng mit dem Zugang zu Informationen und der Agency verwoben ist.

Die Ausbildung einer kritischen Haltung ist im Zusammenhang mit der gesellschaftlichen und institutionellen Organisation des Übergangs in eine Verhütungsbiografie und konkret mit der Einbettung der Verhütungsberatung junger Frauen ins medizinische System zu sehen [10, 27]. Es wurde eine kollektive Praxis der Verschreibung oraler Kontrazeptiva rekonstruiert, die von gesellschaftlichen Normen wie Alters- und Sequenzregeln determiniert ist. Die Analyseergebnisse deuten zudem darauf hin, dass die Ausbildung einer kritischen Haltung nicht notwendigerweise auf die Pille beschränkt bleibt. Die Auswirkungen auf die Verhütungspraxis sind bei den Interviewten unterschiedlich und reichen von einer konsequenten Ablehnung von Hormonen allgemein bis hin zur Nutzung hormoneller Verhütung trotz Unerwünschtheit.

### Limitationen

Die Daten der aktuellen „frauen-leben-4“-Befragung wurden nur in 4 Bundesländern erhoben, womit eine Übertragbarkeit auf Deutschland insgesamt unter Vorbehalt stehen muss. Aufgrund der geänderten Erhebungsmethoden ist die Vergleichbarkeit der Befragungsdaten aus 2012 und 2024 eingeschränkt, von einem prägenden Einfluss auf die sehr deutlichen Ergebnisse des Zeitvergleichs ist jedoch nicht auszugehen. Nicht berücksichtigt ist zudem, inwiefern sich die gynäkologische Verschreibungspraxis hormoneller Kontrazeptiva im betrachteten Zeitraum verändert hat.

Die qualitative Analyse erhebt keinen Anspruch auf Repräsentativität. Als exploratives Verfahren ermöglicht sie, in der Lebenswelt wirksame Strukturen und Wissensbestände systematisch und begründet zu rekonstruieren und damit neue Erklärungsansätze zu gewinnen. Aussagen über die Verbreitung und Reichweite der Analyseergebnisse können und sollen nicht getroffen werden.

## Fazit

Die Ergebnisse der qualitativen Analyse deuten darauf hin, dass Informationsbedarfe zu hormoneller Kontrazeption bei der Beratung in gynäkologischen Praxen gerade zu Beginn der Verhütungsbiografie – aus der Perspektive der Frauen – unzureichend gedeckt werden. Relevant ist dieser Befund, da die gynäkologische Praxis gerade für junge Frauen die wichtigste Informationsquelle darstellt [5]. Die Wirkung einer adäquaten Verhütungsberatung wird auch in der internationalen Forschung betont [29, 30].

Durch ungedeckten Informationsbedarf wird die Selbstbestimmung von Frauen eingeschränkt. Das von Helfferich auf die Gesundheitsversorgung insgesamt gerichtete Diktum: „Wer Selbstbestimmung fördern will, muss … die Verbreitung von notwendigem Wissen absichern“ [28], aufgreifend, unterstreichen die Untersuchungsergebnisse die Bedeutung von bedarfsorientierter Aufklärung und Beratung zur Förderung selbstbestimmter Verhütung.

## Supplementary Information

ESM1: Zusatzmaterial 1

## Footnotes

1 Der alltagssprachliche Begriff „Pille“ wurde in diesem Artikel bewusst gewählt, da sich die Untersuchung primär mit lebensweltlichen Diskursen zu oralen Kontrazeptiva befasst.

2 Agency meint die kognitive Repräsentation der eigenen Handlungs- und Wirkmächtigkeit und umfasst die subjektiven Vorstellungen davon, wer – oder was – wo, wie zum Zustandekommen von Ereignissen beiträgt [16–18].

3 Kurz BIÖG, ehemals Bundeszentrale für gesundheitliche Aufklärung (BZgA).

4 Informationen beziehen sich auf unterschiedliche Quellen, darunter Beratung in gynäkologischen Praxen, eigene oder fremde Erfahrungen mit Nebenwirkungen sowie Diskurse u. a. in sozialen Medien, Fachliteratur und Onlinequellen, die an unterschiedlichen Stellen im Verhütungsprozess zum Tragen kommen.

5 Zur Begriffsbestimmung in der Frauengesundheitsbewegung West siehe Helfferich 2012 [28].
