# Supplementary material for: Decline in the use of hormonal contraceptives among women: genesis of critical attitudes in reproductive biographies: Results from the study “women’s lives 4 — family planning throughout life”
Source: Bundesgesundheitsblatt Gesundheitsforschung Gesundheitsschutz. 2026 Mar 6;69(4):400–9. [Article in German] doi: 10.1007/s00103-026-04211-z (PMC13043558; doi:10.1007/s00103-026-04211-z)
Supplement: Supplementary file 1 — ESM1: Zusatzmaterial 1 [file 103_2026_4211_MOESM1_ESM.pdf]

Onlinematerial

Tabelle Z1 Aktuelle Verhütungsmethoden nach Alter und nach Einstellungen zur Anwendung der Pille. Die Angaben beziehen sich auf Frauen mit Verhütungsbedarf. Datenquelle: Studie „frauen leben 4“ (2024)

	n (ungewichtet, gewichtet)	Pille % [95 % KI]	V; *p*	Kondom % [95 % KI]	V; *p*	Kupferspirale % [95 % KI]	V; *p*	Hormonspirale % [95 % KI]	V; *p*	andere hormonelle Methoden % [95 % KI]	V; *p*
**Gesamt**	5.579, 5.504	25,4 [24,2; 26,5]		45,8 [44,5; 47,1]		5,3 [4,7; 5,9]		6,4 [5,8; 7,0]		2,8 [2,4; 3,3]	
**Altersgruppe**			0,164; 0,000		0,151; 0,000		0,048; 0,012		0,073; 0,000		0,095; 0,000
20 bis 24 Jahre	934, 983	38,5 [35,5; 41,6]		53,2 [50,1; 56,3]		4,8 [3,6; 6,3]		4,5 [3,3; 5,9]		5,0 [3,8; 6,5]	
25 bis 29 Jahre	1.128, 1.058	29,4 [26,7; 32,2]		51,4 [48,4; 54,4]		6,2 [4,9; 7,8]		5,9 [4,6; 7,4]		4,7 [3,6; 6,1]	
30 bis 34 Jahre	1.102, 1.152	21,6 [19,3; 24,1]		50,0 [47,1; 52,9]		6,7 [5,3; 8,2]		5,5 [4,3; 6,9]		2,2 [1,4; 3,1]	
35 bis 39 Jahre	1.195, 1.159	21,3 [19,0; 23,7]		43,1 [40,3; 46,0]		3,7 [2,7; 4,9]		6,0 [4,8; 7,5]		1,4 [0,8; 2,2]	
40 bis 44 Jahre	1.220, 1.153	18,2 [16,1; 20,5]		32,8 [30,1; 35,5]		5,0 [3,9; 6,4]		9,7 [8,1; 11,5]		1,4 [0,8; 2,2]	
**Verhütung mit der Pille hat negative Auswirkungen auf Körper und Seele.**			0,320; 0,000		0,165; 0,000		0,063; 0,000		0,033; 0,051		0,052; 0,000
1-2 (Zustimmung)	3.803, 3.754	16,5 [15,3; 17,7]		51,0 [49,4; 52,6]		6,2 [5,5; 7,0]		5,9 [5,2; 6,7]		2,2 [1,8; 2,7]	
3 (teils-teils)	1.111, 1.131	38,5 [35,7; 41,3]		38,6 [35,8; 41,5]		3,7 [2,7; 4,9]		8,0 [6,5; 9,6]		4,1 [3,0; 5,3]	
4-5 (Ablehnung)	653, 608	56,1 [52,1; 60,0]		27,1 [23,7; 30,8]		2,5 [1,4; 3,9]		6,3 [4,5; 8,4]		4,1 [2,7; 5,9]	
**Verhütung mit der Pille kann man unbedenklich über Jahre hinweg anwenden.**			0,344; 0,000		0,130; 0,000		0,073; 0,000		0,010; 0,758		0,039; 0,017
1-2 (Zustimmung)	859, 920	49,0 [45,8; 52,3]		38,2 [35,1; 41,3]		2,8 [1,9; 4,0]		5,9 [4,5; 7,5]		2,4 [1,5; 3,5]	
3 (teils-teils)	1.195, 1.219	39,9 [37,1; 42,6]		37,4 [34,7; 40,2]		3,6 [2,7; 4,8]		6,4 [5,1; 7,9]		4,0 [3,0; 5,2]	
4-5 (Ablehnung)	3.511, 3.348	13,6 [12,5; 14,8]		51,0 [49,3; 52,7]		6,6 [5,8; 7,5]		6,5 [5,7; 7,4]		2,5 [2,0; 3,1]	
**Für mich persönlich kommt es wegen der Nebenwirkungen nicht (mehr) in Frage, mit der Pille zu verhüten.**			0,698; 0,000		0,200; 0,000		0,148; 0,000		0,046; 0,003		0,066; 0,000
1-2 (Zustimmung)	3.536, 3.380	2,5 [2,1; 3,1]		53,6 [51,9; 55,3]		7,9 [7,0; 8,8]		7,2 [6,3; 8,1]		2,5 [2,0; 3,1]	
3 (teils-teils)	481, 581	36,7 [32,8; 40,6]		38,7 [34,8; 42,7]		1,9 [1,0; 3,3]		6,7 [4,9; 9,0]		6,0 [4,3; 8,2]	
4-5 (Ablehnung)	1.550, 1.538	71,2 [68,9; 73,5]		31,5 [29,2; 33,8]		0,8 [0,5; 1,4]		4,6 [3,7; 5,8]		2,4 [1,7; 3,3]	

Tabelle 4 (Fortsetzung)

Aktuelle Verhütungsmethoden nach Alter und nach Einstellungen zur Anwendung der Pille. Die Angaben beziehen sich auf Frauen mit Verhütungsbedarf. Datenquelle: Studie „frauen leben 4“ (2024)

	**n (ungewichtet, gewichtet)**	**zyklusbasierte Methoden % [95 % KI]**	**V; *p***	**Sterilisation (Befragte/ Partner) % [95 % KI]**	**V; *p***	**Coitus Interruptus % [95 % KI]**	**V; *p***	**Sonstiges % [95 % KI]**	**V; *p***	**keine Verhütung % [95 % KI]**	**V; *p***
**Gesamt**	5.579, 5.504	9,7 [8,9; 10,5]		5,9 [5,3; 6,5]		5,6 [5,0; 6,3]		6,3 [5,7; 7,0]		6,7 [6,1; 7,4]	
**Altersgruppe**			0,046; 0,020		0,235; 0,000		0,015; 0,867		0,045; 0,024		0,073; 0,000
20 bis 24 Jahre	934, 983	8,9 [7,2; 10,8]		0,3 [0,1; 0,8]		5,5 [4,2; 7,1]		6,4 [5,0; 8,1]		3,4 [2,4; 4,6]	
25 bis 29 Jahre	1.128, 1.058	10,6 [8,8; 12,6]		0,9 [0,5; 1,7]		6,1 [4,8; 7,7]		4,4 [3,2; 5,7]		6,9 [5,5; 8,6]	
30 bis 34 Jahre	1.102, 1.152	11,2 [9,5; 13,1]		3,0 [2,2; 4,2]		5,8 [4,6; 7,3]		7,6 [6,2; 9,3]		6,1 [4,8; 7,6]	
35 bis 39 Jahre	1.195, 1.159	10,1 [8,5; 11,9]		9,0 [7,4; 10,7]		5,1 [3,9; 6,5]		6,2 [4,9; 7,7]		8,4 [6,9; 10,1]	
40 bis 44 Jahre	1.220, 1.153	7,5 [6,0; 9,1]		14,7 [12,8; 16,9]		5,6 [4,4; 7,1]		7,0 [5,7; 8,6]		8,5 [7,0; 10,2]	
**Verhütung mit der Pille hat negative Auswirkungen auf Körper und Seele.**			0,141; 0,000		0,033; 0,049		0,067; 0,000		0,009; 0,782		0,065; 0,000
1-2 (Zustimmung)	3.803, 3.754	12,4 [11,4; 13,5]		5,5 [4,8; 6,3]		6,7 [5,9; 7,5]		6,5 [5,7; 7,3]		7,8 [7,0; 8,7]	
3 (teils-teils)	1.111, 1.131	4,2 [3,2; 5,5]		5,8 [4,5; 7,2]		4,0 [3,0; 5,2]		6,0 [4,7; 7,5]		5,4 [4,2; 6,8]	
4-5 (Ablehnung)	653, 608	2,3 [1,3; 3,7]		8,1 [6,1; 10,4]		2,5 [1,4; 3,9]		5,9 [4,3; 8,0]		3,0 [1,8; 4,5]	
**Verhütung mit der Pille kann man unbedenklich über Jahre hinweg anwenden.**			0,135; 0,000		0,020; 0,340		0,060; 0,000		0,049; 0,001		0,045; 0,003
1-2 (Zustimmung)	859, 920	4,8 [3,5; 6,3]		6,0 [4,6; 7,7]		3,6 [2,5; 4,9]		3,8 [2,7; 5,2]		4,2 [3,1; 5,7]	
3 (teils-teils)	1.195, 1.219	4,6 [3,5; 5,9]		5,0 [3,9; 6,3]		4,2 [3,2; 5,4]		5,9 [4,7; 7,3]		6,9 [5,6; 8,4]	
4-5 (Ablehnung)	3.511, 3.348	12,8 [11,7; 14,0]		6,2 [5,4; 7,0]		6,8 [5,9; 7,6]		7,0 [6,2; 7,9]		7,4 [6,5; 8,3]	
**Für mich persönlich kommt es wegen der Nebenwirkungen nicht (mehr) in Frage, mit der Pille zu verhüten.**			0,173; 0,000		0,097; 0,000		0,111; 0,000		0,028; 0,122		0,124; 0,000
1-2 (Zustimmung)	3.536, 3.380	13,6 [12,5; 14,8]		7,6 [6,8; 8,6]		6,7 [5,9; 7,5]		6,7 [5,9; 7,6]		9,1 [8,1; 10,1]	
3 (teils-teils)	481, 581	6,4 [4,6; 8,6]		3,3 [2,0; 5,0]		9,8 [7,6; 12,4]		6,9 [5,0; 9,2]		5,9 [4,2; 8,0]	
4-5 (Ablehnung)	1.550, 1.538	2,2 [1,6; 3,0]		2,9 [2,1; 3,8]		1,8 [1,2; 2,6]		5,3 [4,2; 6,5]		2,0 [1,4; 2,8]	
